# Wheat Bran Does Not Affect Postprandial Plasma Short-Chain Fatty Acids from ^13^C-inulin Fermentation in Healthy Subjects

**DOI:** 10.3390/nu9010083

**Published:** 2017-01-20

**Authors:** Lise Deroover, Joran Verspreet, Anja Luypaerts, Greet Vandermeulen, Christophe M. Courtin, Kristin Verbeke

**Affiliations:** 1 Translational Research in Gastrointestinal Disorders, KU Leuven, Leuven 3000, Belgium; lise.deroover@kuleuven.be (L.D.); anja.luypaerts@kuleuven.be (A.L.); greet.vandermeulen@kuleuven.be (G.V.); 2 Centre for Food and Microbial Technology, KU Leuven, Leuven 3000, Belgium; joran.verspreet@kuleuven.be (J.V.); christophe.courtin@kuleuven.be (C.M.C.); 3 Leuven Food Science and Nutrition Research Centre, KU Leuven, Leuven 3000, Belgium

**Keywords:** colonic fermentation, short-chain fatty acids, wheat bran, inulin

## Abstract

Wheat bran (WB) is a constituent of whole grain products with beneficial effects for human health. Within the human colon, such insoluble particles may be colonized by specific microbial teams which can stimulate cross-feeding, leading to a more efficient carbohydrate fermentation and an increased butyrate production. We investigated the extent to which WB fractions with different properties affect the fermentation of other carbohydrates in the colon. Ten healthy subjects performed four test days, during which they consumed a standard breakfast supplemented with 10 g ^13^C-inulin. A total of 20 g of a WB fraction (unmodified WB, wheat bran with a reduced particle size (WB RPS), or de-starched pericarp-enriched wheat bran (PE WB)) was also added to the breakfast, except for one test day, which served as a control. Blood samples were collected at regular time points for 14 h, in order to measure ^13^C-labeled short-chain fatty acid (SCFA; acetate, propionate and butyrate) concentrations. Fermentation of ^13^C-inulin resulted in increased plasma SCFA for about 8 h, suggesting that a sustained increase in plasma SCFA can be achieved by administering a moderate dose of carbohydrates, three times per day. However, the addition of a single dose of a WB fraction did not further increase the ^13^C-SCFA concentrations in plasma, nor did it stimulate cross-feeding (Wilcoxon signed ranks test).

## 1. Introduction

The short-chain fatty acids (SCFA) acetate, propionate, and butyrate, constitute a major class of bacterial metabolites that are derived from colonic carbohydrate fermentation. They are increasingly considered as signaling molecules with a beneficial impact on gut and systemic health [1,2]. Indeed, besides serving as energy substrates for the colonocytes, SCFA influence the expression of many genes by acting as inhibitors of histone deacetylases, and affect metabolic processes through the activation of G-protein coupled receptors (GPR41 and GPR43, later renamed as free fatty acid receptor (FFAR)-3 and (FFAR)-2). Several studies have indicated that SCFA, and in particular butyrate, improve the intestinal barrier function and reduce inflammation by inhibiting NFκB activation [3,4]. In addition, SCFA that enter the systemic circulation modulate energy homeostasis, and peripheral glucose and lipid metabolism [4,5,6]. Furthermore, SCFA regulate immune function by affecting T cell differentiation into effector and regulatory T cells (Treg) [7], and colonic Treg cell homeostasis [8].

As a consequence, strategies that target the microbiota to improve health often aim at increasing saccharolytic fermentation. The production of colonic SCFA may be stimulated by modulating the intestinal microbiota and increasing the numbers of acetate and butyrate producing bacteria via administration of probiotics. Many probiotics are selected strains of lactobacilli or bifidobacteria [9], which are known lactate and acetate producers. In addition, there is a growing interest in the use of butyrate producing bacteria, such as *Faecalibacterium prausnitzii* and *Butyrcicoccus pullicaecorum*, as probiotics [10,11,12,13].

An alternative strategy for increasing SCFA production is to stimulate the indigenous saccharolytic bacterial population by administration of prebiotics or fermentable dietary fibers [14]. The best studied prebiotic substrates are inulin-type fructans (comprising oligofructose), galacto-oligosaccharides, xylo-oligosaccharides, and arabinoxylanoligosaccharides. In vitro fermentation studies with fecal inocula indicate that the amount and proportion of SCFA produced, depends on the type of fermentable substrate [15]. For example, inulin-type fructans induce a relatively high proportion of acetate, whereas resistant starch favors butyrate production [16].

Here, we applied a third strategy for modulating the entire intestinal ecosystem, which involves the administration of disperse insoluble particles that act as platforms on which the bacteria can adhere, grow, and interact. We hypothesize that these particles facilitate the exchange of microbial nutrients and metabolites, resulting in a more complete carbohydrate fermentation and increased cross-feeding. For example, the production of butyrate requires collaboration between primary degraders, such as bifidobacteria, that produce acetate and lactate, and butyrate producing bacteria, such as *Faecalibacterium* and *Roseburia*, that convert acetate into butyrate [17,18]. This interaction may be facilitated if both species adhere to the dietary platforms. Wheat bran (WB) was selected as an interesting dietary component for this purpose, as bacterial communities attached to WB that was incubated in vitro with human fecal inocula, were found to be dominated by *Clostridium* cluster XIVa bacteria, known as butyrate producers [19]. In addition, WB can be easily technically modified to control its physical properties. In this study, we evaluated the impact of three WB fractions that differed in particle size and tissue composition, on the fermentation of a readily fermentable carbohydrate (^13^C-inulin) in healthy subjects. Concentrations of ^13^C-SCFA were measured in plasma as an indication of carbohydrate fermentation and the relative proportions of acetate, propionate, and butyrate, were considered as a marker of cross-feeding.

## 2. Materials and Methods

### 2.1. WB Fractions

#### 2.1.1. Unmodified WB

Commercial WB with a particle size of 1690 µm was obtained from Dossche Mills (Deinze, Belgium) and was used without further modification. Its chemical composition (amounts of dietary fiber, starch, protein, lipid, and ash) was analysed as previously described [20,21,22].

#### 2.1.2. Wheat Bran with Reduced Particle Size (WB RPS)

The unmodified commercial WB mentioned above was milled in a Cyclotec 1093 Sample mill (FOSS, Höganäs, Sweden), as described previously [22], in order to obtain WB particles with an average size of 150 µm.

#### 2.1.3. Destarched Pericarp-Enriched Wheat Bran (PE WB)

PE WB was ascertained from Fugeia N.V. (Leuven, Belgium) and was obtained after an amylase and xylanase treatment of untreated WB, as described by Swennen et al. [23]. Subsequently, the PE WB was reduced in particle size to about 280 µm, using the same method as mentioned above.

### 2.2. Fermentable Substrate

Highly ^13^C-enriched inulin with an atom percent (AP) beyond 97% was purchased from Isolife (Wageningen, The Netherlands) and was mixed with unlabeled native inulin (Fibruline instant, Cosucra Groupe Warcoing SA, Warcoing, Belgium; AP 0.98%), to form a homogeneous mixture with an AP of 1.93%.

### 2.3. Study Population

Ten healthy men and woman, aged between 18 and 65 years, were recruited to participate in the study. All subjects had a body mass index (BMI) between 18 and 27 kg/m^2^ and a regular diet defined as three meals per day, on at least five days per week. Exclusion criteria were the use of antibiotics, prebiotics and probiotics, in the month preceding the study and during the study, consumption of a low calorie diet or another special diet in the month prior to the study, the use of medication that could affect the gastrointestinal tract in the two weeks before the start of the study and during the study, abdominal surgery in the past (except for appendectomy), chronic gastrointestinal diseases, blood donation in the three months prior to the study, hemoglobin (Hb) levels below reference values, and for woman, pregnancy or breast feeding. Subjects that had participated in a clinical trial involving radiation exposure in the year prior to the study were also excluded. The study protocol conformed to the Declaration of Helsinki and was approved by the Ethics Committee of the University of Leuven (Belgian Registration Number: B322201423101). All participants signed written informed consent. The study has been registered at ClinicalTrials.gov (clinical trial number: NCT02422537).

### 2.4. Study Design

**Figure 1:**
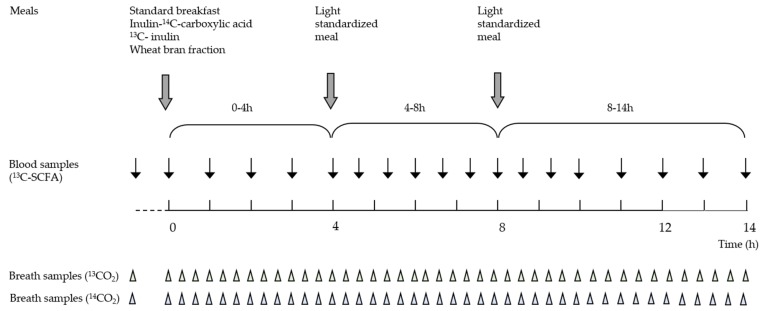
After a standard breakfast, labeled with ^13^C-inulin, inulin-^14^C-carboxylic acid and a WB fraction, breath samples were collected for the measurement of ^13^CO_2_ and ^14^CO_2_ and blood samples were collected for the analysis of ^13^C-SCFA concentrations for 14 h. A light standardized meal was administered after 4 h and 8 h. The participants were blind to the order of the different WB fractions and control tests, which were randomized.

Each subject performed four test days, with at least one week in between each test. During the three days prior to each test day, subjects were instructed to consume a low fiber diet, consisting of a maximum of one piece of fruit per day, white bread instead of wholegrain products, and no more than 100 g vegetables per day. They were also asked to avoid alcohol consumption. On the evening prior to the test day, the subjects consumed a completely digestible and non-fermentable meal (lasagna), eventually supplemented with white bread. After an overnight fast, the subjects presented themselves at the laboratory and provided two basal breath samples for the measurement of ^13^CO_2_ and ^14^CO_2_. A catheter (BD, Erembodegem, Belgium) was placed into an antecubital vein in the forearm to collect all blood samples during the test day. After collection of a basal blood sample, a standard breakfast was administered to the subjects. The breakfast consisted of 250 g low-fat yoghurt labeled with inulin-^14^C-carboxylic acid (74 kBq, Perkin Elmer, Boston, MA, USA), a marker for oro-cecal transit time (OCTT), and 10 g of ^13^C-labeled inulin, which served as a model fermentable substrate. The OCTT was defined as the time that elapses between the intake of the meal, and the arrival in the colon, which is reflected by the appearance of ^14^CO_2_ in the breath. Depending on the test day, the breakfast was further supplemented with 20 g of one of the three WB fractions, or no supplement on the control test day. The participants were blind to the order of the different WB fractions and control tests, which were randomized using online software [24]. Breath samples were collected every 20 min, up to 14 h after consumption of the breakfast. Blood samples were collected every hour during the first 4 h, every 40 min from 4 h to 10 h, and again every hour from 10 h to 14 h. A light digestible meal was offered to the subjects 4 h and 8 h after consumption of the breakfast. Water was offered *ad libitum* during the whole test day. The course of a test day is represented in Figure 1.

### 2.5. Collection and Analysis of Breath Samples

Subjects deposited breath samples for the analysis of ^13^CO_2_ by blowing through a straw, into a 12-mL glass tube (Exetainer^®^, Labco Ltd., Ceredigion, UK), whereas samples for the measurement of ^14^CO_2_ were collected by blowing through a pipet, into a plastic scintillation vial (Sarstedt, Nümbrecht, Germany) containing 4 mL of a 0.5 M hyamine hydroxide solution (Perkin Elmer, Boston MA, USA). Thymolphtaleine acted as a color indicator, which became discolored when 2 mmol CO_2_ was exhaled.

The abundance of ^13^CO_2_ was measured using isotope ratio mass spectrometry (ABCA, Sercon, Crewe, UK) and the results were expressed as delta over baseline (DOB). ^14^CO_2_ was measured using β-scintillation counting (Packard Tricarb Liquid Scintillation Spectrometer, model 3375, Packard Instruments, Downers Grove, IL, USA), after addition of 10 mL hionic fluor (Perkin Elmer, Boston MA, USA), and was expressed as disintegrations per minute (DPM) [25]. The arrival time of the breakfast in the colon (OCTT) and the start of the fermentation, were defined as the time at which a significant increase in ^14^CO_2_ and ^13^CO_2_, respectively, from the background was observed in the breath. This increase was defined as 2.5 times the standard deviation of all previous points, above the running average of all previous points [26].

### 2.6. Analysis of Plasma ^13^C-Abundance

Blood samples were centrifuged at 3000× *g* and 4 °C for 10 min to obtain plasma. The samples were immediately aliquoted and stored at −80 °C until analysis. Plasma samples were deproteinized using Amicon^®^ Ultra-15 filters (Molecular weight cut-off: 30 kDa, Merck, Kenilworth, NJ, USA). Before application of the sample, the filters were rinsed twice using 0.2 N HCl to avoid SCFA contamination. Subsequently, 3 mL plasma was mixed with 3 mL MilliQ^®^ water (Sartorius Arium^®^ 611-VF, Sartorius, Göttingen, Germany) and 150 µL 0.15 N NaOH, and centrifuged at 2000× *g* and 4 °C for 20 min. Following this, the samples were transferred to the cleaned filters and deproteinized by centrifugation at 3000× *g* and 4 °C for 3 h. After addition of 120 µL 1 M NaOH, the filtrate was dried overnight in a vacuum concentrator (RVC 2-18, Christ, Germany) at 50 °C.

Prior to injection, the dried samples were acidified with 100 µL 4 M HCl with trypan blue (0.4%, Sigma, UK), and extracted in 400 µL diethyl ether (Sigma-Aldrich, Steinheim, Germany). The ether layer was pipetted into a crimp neck vial (1.5 mL) and evaporated with nitrogen gas (N_2_) to about 50 µL, before injection in the gas chromatograph combustion isotope ratio mass spectrometer (GC-C-IRMS) (Delta plus-XP, Thermo Fisher, Bremen, Germany), equipped with a trace gas chromatograph (Interscience, Breda, The Netherlands) and a combustion interface type 3 (Thermo Fisher). Four µL of the SCFA solution was injected on an AT-Aquawax-DA column (30 m × 0.53 mm, i.d., 1.00 µm, Grace, Lokeren, Belgium), with the injector temperature at 240 °C. Helium 5.0 was used as a carrier gas at a constant flow of 2.5 mL/min. The initial oven temperature was held at 80 °C for 3 min and was increased by 4 °C/min to 140 °C, then further increased by 8 °C/min to 240 °C, and kept at this temperature for 10 min. The separated GC-effluents were combusted and oxidised to NO_x_, CO_2_, and H_2_O in an oxidation furnace (CuO/NiO/Pt) at 940 °C [25]. All oxidised components were passed over the reduction column at 640 °C and the H_2_O in the system was eliminated by a Nafion membrane (Thermo Fisher). The delta (δ^13^ Pee Dee Belemnite (PDB)) values were calculated by Isodat 2.0 software (Thermo Fisher) and converted to AP. The measured AP at any time point *t* was subtracted from the abundance measured in the baseline sample at time point 0, to obtain results in atom per cent excess (APE) [25]. The linearity of the system (slope < 0.06) was confirmed to be in the range between 0.8 and 11 volts, with CO_2_ as the reference gas (5.0 quality, δ = −32.16‰).

### 2.7. Analysis of Total SCFA Concentrations in Plasma

The total concentration of acetate, propionate, and butyrate in the plasma was measured using gas chromatography coupled with a flame ionization detector (GC-FID) after preconcentration of the SCFA, using a hollow fiber liquid membrane extraction. Plasma samples were thawed prior to analysis, and prepared and analyzed as described by Zhao et al. [27], with slight modifications. Plasma samples (100 µL) were rapidly spiked with internal standard (12.5 µg 2-ethyl butyric acid and 30 µg 3-methyl-valeric acid), acidified with 20 µL 0.2 N HCl, and diluted to 1.5 mL. A hollow fiber coated with tri-n-octylphoshphine oxide and filled with 10 µL 0.15 N NaOH, was immersed in the diluted plasma and shaken overnight. In this way, protonated SCFA diffuse into the fiber, where they become ionized and remain trapped. After acidification of the fiber content, 0.5 µL of the acidified SCFA solution was injected in a GC (HP 6890 series, Agilent, Wilmington, NC, USA) equipped with a FID and a DB-FAPP capillary column (30 m × 0.53 mm id, 1.00 µm film, Agilent, Wilmington, NC, USA). Helium was used as a carrier gas at a flow rate of 4.2 mL/min. The initial oven temperature was 100 °C for 3 min, raised by 4 °C/min to 140 °C, and held at this temperature for 5 min, before being further increased by 40 °C/min to 235 °C, and finally held at 235 °C for 5 min. The temperature of the FID heater and the injection port were set at 240 °C and 200 °C, respectively. The flow rates of hydrogen, air, and nitrogen as the make-up gas, were 30, 300, and 20 mL/min, respectively.

### 2.8. Calculations

At each time point, the concentration of ^13^C-SCFA in plasma is the sum of the concentration that was already present at the baseline, and the concentration that originates from the colon (Equation (1)).

(1) [C-SCFA13] = [C-SCFA13]t0 + [C-SCFA13]colon

where [C-SCFA13]t0 is the concentration of ^13^C-SCFA present in plasma at time point 0 and [C-SCFA13]colon is the concentration of ^13^C-SCFA coming from the colon at time point t.

Substitution of the concentration of ^13^C-SCFA by the product of its total concentration and abundance, results in Equation (2).

(2) $$n_{t}\;\times \;{\mathrm{AP}}_{t}\;=\;n_{0}\;\times \;{\mathrm{AP}}_{{\mathrm{plasma}}_{t_{0}}}\;+\;n_{\mathrm{colon}}\;\times \;{\mathrm{AP}}_{\mathrm{colon}}$$

In addition, the total concentration of SCFA at each time point t is the sum of the SCFA already present in the plasma, and the SCFA produced in the colon (Equation (3)).

(3) $$n_{t}\;=\;n_{0}\;+\;n_{\mathrm{colon}}$$

Substitution of $n_{0}$ by $n_{t}$-$n_{\mathrm{colon}}$ in Equation (2), allows one to calculate the concentration of SCFA originating from the colon, thus produced from the ^13^C-inulin at a time point t, according to Equation (4).

(4) $$n_{\mathrm{colon}}\;=\;n_{t}\;\times \;\frac{\left({\mathrm{AP}}_{t}\;-{\mathrm{AP}}_{{\mathrm{plasma}}_{t_{0}}}\right)}{\left({\mathrm{AP}}_{\mathrm{colon}}-{\mathrm{AP}}_{{\mathrm{plasma}}_{t_{0}}}\right)}$$

where $n_{\mathrm{colon}}$ is the concentration of SCFA originating from the colon; $n_{t}$ is the total concentration of SCFA at time point t; ${\mathrm{AP}}_{t}$ is the AP of ^13^C-acetate, ^13^C-propionate, or ^13^C-butyrate at time point t; ${\mathrm{AP}}_{\mathrm{colon}}$ is the AP of the administered inulin; ${\mathrm{AP}}_{{\mathrm{plasma}}_{t_{0}}}$ is the atom percent of ^13^C-acetate, ^13^C-propionate, or ^13^C-butyrate in the plasma at time point $t_{0}$.

The concentrations of ^13^C-acetate, ^13^C-propionate, and ^13^C-butyrate, originating from the colon at each time point, were used to draw concentration versus time curves for each test day. Subsequently, the cumulative concentrations of ^13^C-acetate, ^13^C-propionate, and ^13^C-butyrate (area under the curve (AUC)) were calculated using the trapezoidal rule and were expressed in μmol·h/L.

The starting point of the fermentation was ascertained by calculating the mean time at which ^13^C-acetate, ^13^C-propionate, and ^13^C-butyrate concentrations in plasma increased. The end of the fermentation was the mean time at which ^13^C-acetate, ^13^C-propionate, and ^13^C-butyrate concentrations returned to the baseline. The duration of the fermentation was the difference between the end and start points of the fermentation.

### 2.9. Statistical Analysis

Statistical analysis was performed using SPSS software, version 23.0 (IBM, Brussels, Belgium). Because of the small sample size, non-parametric tests were used (Wilcoxon signed ranks test).

## 3. Results

### 3.1. Characterisation of the WB Fractions

**Figure 2:**
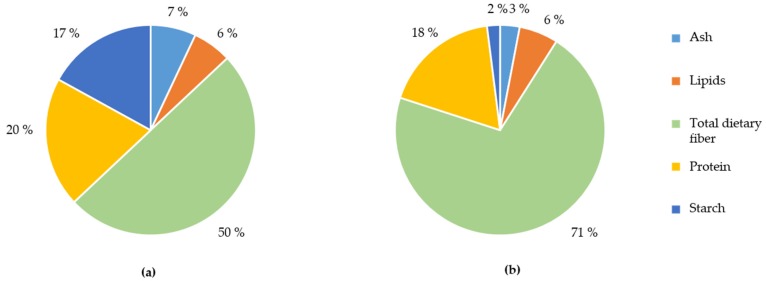
Unmodified wheat bran (WB) and wheat bran with reduced particle size (WB RPS) (**a**) contain less total dietary fiber and more starch compared to destarched pericarp-enriched wheat bran (PE WB) (**b**).

Untreated WB had an average particle size of 1690 µm and was composed of 50% dietary fiber (arabinoxylan, cellulose, β-glucan, lignin and fructan), 17% starch, 20% protein, 6% lipids, and 7% ash (Figure 2a). WB RPS did not differ in tissue composition from the unmodified WB (Figure 2a). In contrast, PE WB was composed of 71% dietary fiber (mainly insoluble), 2% starch, 18% protein, 6% lipids, and 7% ash (Figure 2b).

### 3.2. Study Population

**Figure 3:**
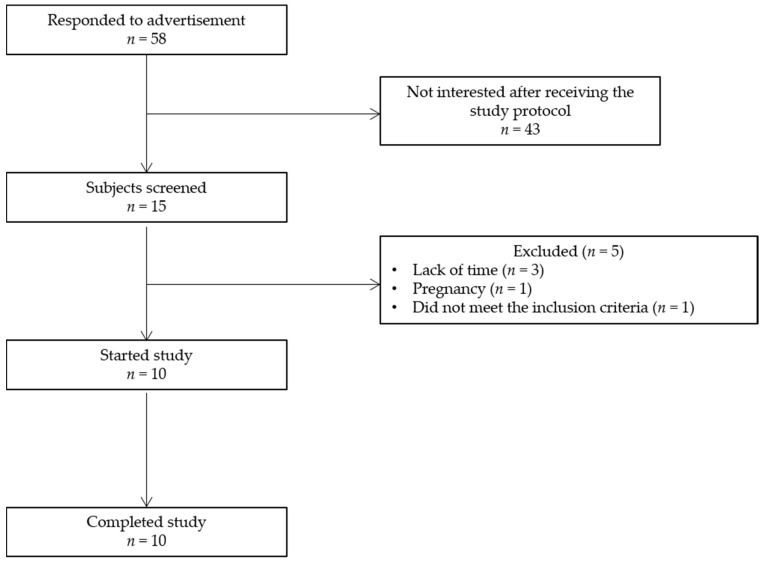
Flow chart depicting the passage of subjects through the study.

From the 58 subjects that responded to the advertisement, 15 subjects underwent screening, which included an assessment of height and body weight, plasma Hb levels, medical history, and dietary habits (Figure 3). Five volunteers withdrew from the study before the start because of a lack of time (*n* = 3), pregnancy (*n* = 1), or because they did not meet the inclusion criteria (*n* = 1). Ten subjects (4M/6F, aged 25 ± 4 years, BMI 23.7 ± 2.0 kg/m^2^) completed the four test days according to the study protocol.

### 3.3. Estimation of the OCTT and the Start of the Fermentation Using Breath and Plasma Samples

**Figure 4:**
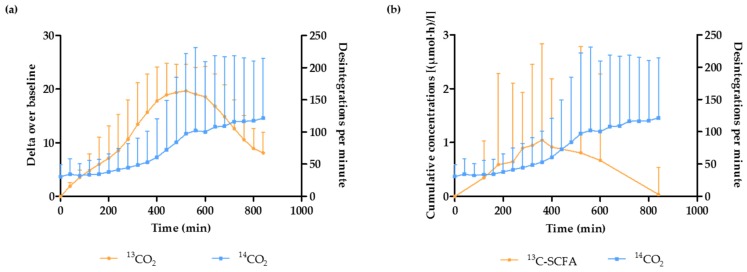
Both ^13^C-excretion in breath (**a**) and plasma ^13^C-SCFA concentrations (**b**) start to increase before the increase in breath ^14^CO_2_ (*n* = 40).

The start of the fermentation was defined as the time point at which ^13^CO_2_ started to increase in the breath, or as the mean time point at which the concentration of ^13^C-acetate, ^13^C-propionate, and ^13^C-butyrate started to increase in the plasma. An increase in breath ^14^CO_2_, which was generated from bacterial fermentation of ^14^C-labeled inulin carboxylic acid, was used as a marker for the OCTT. Figure 4a,b compare the OCTT to the start of fermentation, based on breath ^13^CO_2_ excretion and plasma ^13^C-SCFA concentrations, respectively. The increase in breath ^13^CO_2_ and in plasma ^13^C-SCFA occurred slightly before the increase in breath ^14^CO_2_, suggesting that fermentation of ^13^C-inulin had already started in the terminal ileum.

**Figure 5:**
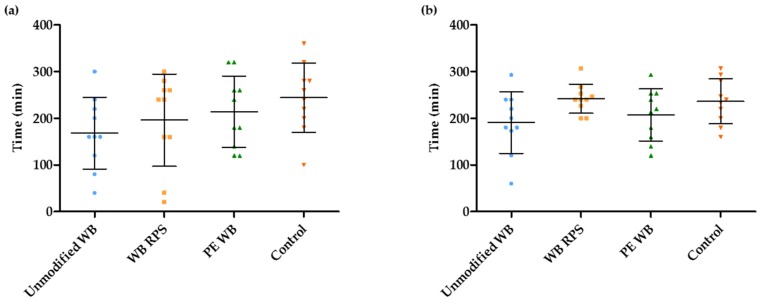
Wheat bran (WB) supplementation did not affect the start of the fermentation based on the ^13^CO_2_-breath excretion (**a**) nor ^13^C-labeled short-chain fatty acids (SCFA) concentrations in plasma (**b**) (*n* = 10). The middle line represents the mean with the standard deviation (whiskers).

On average, the fermentation started 208.5 ± 29 min and 219 ± 24.2 min after consumption of the breakfast, based on the breath samples and the plasma samples, respectively. The start of the fermentation was not affected by the addition of any of the WB fractions, when compared to the control (Wilcoxon signed ranks test; Breath samples: p_WB_ = 0.073, p_WBRPS_ = 0.125, p_PEWB_ = 0.619; Plasma samples: p_WB_ = 0.123, p_WBRPS_ = 0.482, p_PEWB_ = 0.066) (Figure 5a,b).

### 3.4. Estimation of the Duration of the Fermentation in the Presence of Different WB Fractions

**Figure 6:**
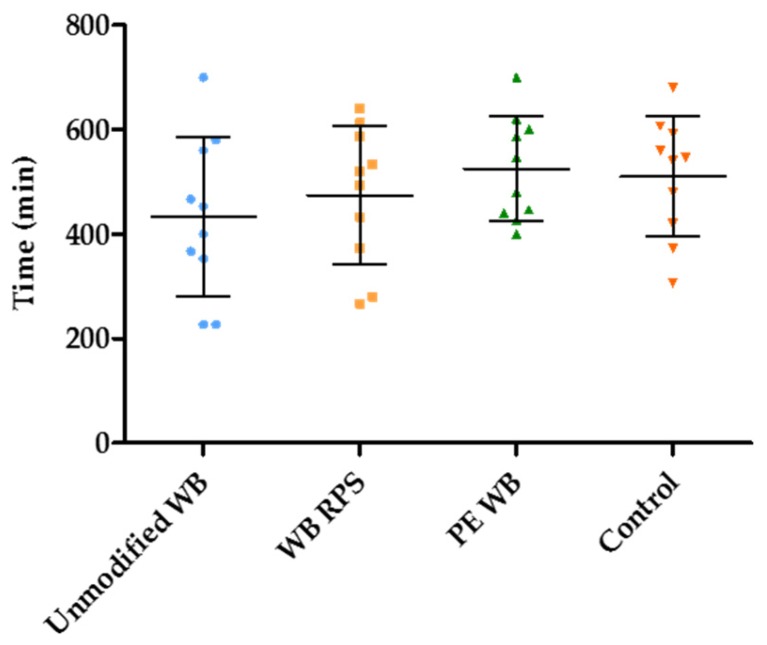
Wheat bran (WB) supplementation did not modify the duration of the fermentation (*n* = 10). The middle line represents the mean with the standard deviation (whiskers).

The duration of the fermentation was considered as a measure for the efficiency of fermentation with shorter duration, indicating a more efficient fermentation. On average, fermentation continued for 486 ± 40.9 min. However, the duration of the fermentation was not different when unmodified WB, WB RPS, and PE WB were administered to the subjects, compared to the control condition (Wilcoxon signed rank test; p_WB_ = 0.201, p _WBRPS_ = 0.236, p_PEWB_ = 0.878) (Figure 6).

### 3.5. ^13^C-SCFA Concentrations in Plasma Produced from the ^13^C-Labeled Inulin

**Figure 7:**
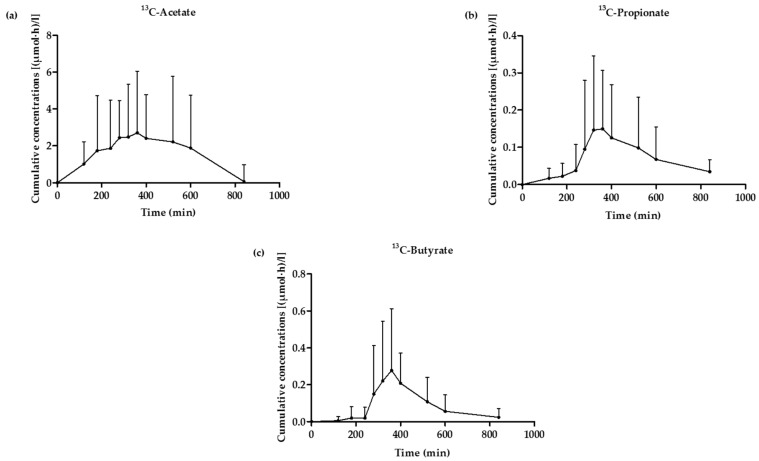
Average plasma concentrations of ^13^C-acetate (**a**), ^13^C-propionate (**b**) and ^13^C-butyrate (**c**) originating from the colon as a function of time (*n* = 40).

Average concentrations of plasma ^13^C-acetate, ^13^C-propionate, and ^13^C-butyrate originating from the colon, were presented as a function of time (Figure 7).

**Figure 8:**
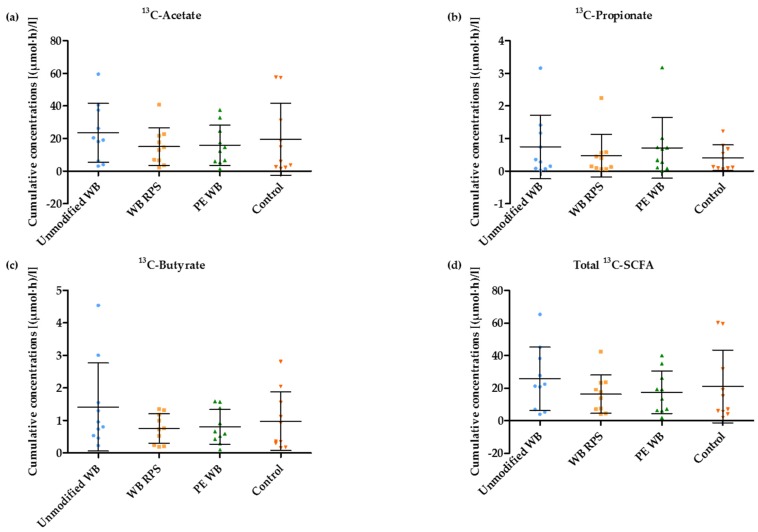
Wheat bran (WB) supplementation did not affect the cumulative ^13^C-acetate (**a**), ^13^C-propionate (**b**), ^13^C-butyrate (**c**), total ^13^C-SCFA (**d**) concentrations in plasma (*n* = 10). The middle line represents the mean with the standard deviation (whiskers).

Influx of ^13^C-acetate, ^13^C-propionate, and ^13^C-butyrate from the colon, reached a maximum value 360 ± 119 min after consuming the breakfast. Mean cumulative ^13^C-SCFA concentrations (*n* = 40) amounted to 18.5 ± 3.88 µmol·h·L^−1^ for acetate, 0.59 ± 0.17 µmol·h·L^−1^ for propionate, and 0.99 ± 0.3 µmol·h·L^−1^ for butyrate. However, cumulative ^13^C-SCFA plasma concentrations were not different in the presence of any WB fraction, when compared to the control condition (Wilcoxon signed ranks test; Acetate: p_WB_ = 0.114, p_WBRPS_ = 0.878, p_PEWB_ = 0.721; Propionate: p_WB_ = 0.169, p_WBRPS_ = 0.646, p_PEWB_ = 0.139; Butyrate: p_WB_ = 0.169, p_WBRPS_ = 0.721, p_PEWB_ = 0.646; Total SCFA: p_WB_ = 0.074, p_WBRPS_ = 0.799, p_PEWB_ = 0.646) (Figure 8).

### 3.6. Relative Proportion of Acetate, Propionate, and Butyrate after WB Supplementation

**Figure 9:**
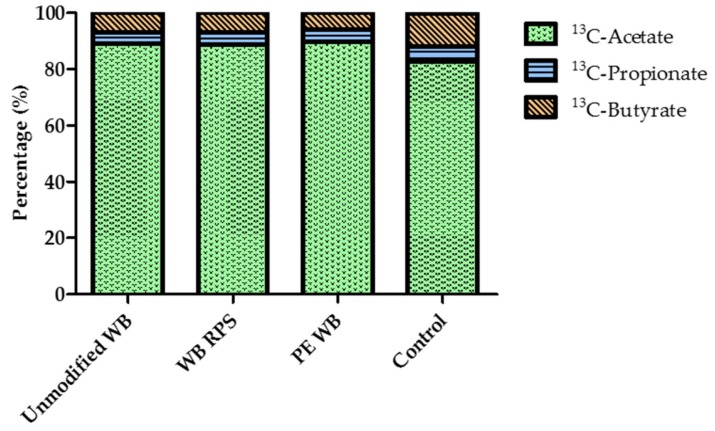
The proportions of ^13^C-acetate, ^13^C-propionate, and ^13^C-butyrate in plasma were not affected by wheat bran (WB) (*n* = 10).

Stimulation of cross-feeding between colonic bacteria was evaluated by calculating the relative proportion of acetate, propionate, and butyrate for the different conditions. However, none of the WB fractions significantly influenced the relative proportion of acetate, propionate, and butyrate in the plasma, when compared to the control (Wilcoxon signed rank test; Acetate: p_WB_ = 0.721, p_WBRPS_ = 0.959, p_PEWB_ = 0.575; Propionate: p_WB_ = 0.959, p_WBRPS_ = 0.445, p_PEWB_ = 0.959; Butyrate: p_WB_ = 0.575, p_WBRPS_ = 0.799, p_PEWB_ = 0.799 (Figure 9).

## 4. Discussion

Colonic production of SCFA from undigested carbohydrates has been increasingly recognized as a key process that contributes to both local gut and systemic health. The extent to which colonic-derived SCFA reach the systemic circulation may be an important parameter that determines the systemic health effects induced by dietary fiber consumption [25]. In the present study, we demonstrated that plasma SCFA are temporarily increased after consumption of a moderate dose of an easily fermentable carbohydrate. 

The fact that we used uniformly stable isotope labeled inulin which, upon fermentation, resulted in the formation of labeled SCFA, allowed us to selectively quantify in plasma those SCFA originating from the inulin fermentation in the colon and to exclude confounders, like SCFA that were present in the colon at the start of the test days, SCFA that might have been produced due to partial fermentation of the WB fractions, or SCFA that have been endogenously produced. For example, plasma acetate is also produced from fatty acid oxidation and amino acid metabolism [28], and during ketogenesis in the mitochondria of the hepatocytes [29]. Colonic fermentation of ^13^C-inulin contributed to increased plasma ^13^C-SCFA concentrations for about 8 h, with maximal concentrations 6 h after consumption of the carbohydrate. It needs to be mentioned that the peak SCFA concentrations depend on the transit time of the substrates through the gastrointestinal tract, which is, apart from host factors, determined by the composition and caloric load of the breakfast meal and may vary with another test meal. Nevertheless, the results suggest that sustained increased plasma levels of SCFA might be obtained by three administrations of a considerable dose of an easily fermentable carbohydrate, evenly spread over a 24-h period. Furthermore, experiments which evaluate the health benefits of fermentable carbohydrates or prebiotics that are attributed to systemic SCFA, should preferably be performed in a time window between 6 and 10 h after administration of the carbohydrate.

To investigate whether insoluble particles could stimulate the fermentation of other carbohydrates in the colon, we used WB fractions that differed in tissue composition and particle size. Although reduction of particle size does not alter the composition of the WB fraction, it clearly modifies the physical properties of the WB. Destruction of cell walls during milling increases the specific surface area and the accessibility of intracellular cell components to degrading enzymes and, in this way, augments the fermentability of the WB fractions. In contrast, PE WB has been stripped of any fermentable material and mainly contains highly cross-linked dietary fiber that is hardly fermentable. We hypothesized that PE WB might facilitate fermentation by providing a platform on which bacteria can adhere, resulting in a more efficient exchange of microbial nutrients and metabolites between different types of bacteria; so-called cross-feeding. RPS WB may act in the same way, but has the additional advantage of being partly fermentable. If we would compare PE WB with a dinner table to which the bacteria draw up, WB RPS would be a dinner table filled with nutrients. Hence, we expected WB RPS to be more efficient in stimulating carbohydrate fermentation and cross-feeding.

Several factors may explain why WB did not stimulate the fermentation of inulin. First, inulin is an easily and rapidly fermentable substrate for colonic bacteria. It is possible that the fermentation proceeds so efficiently in baseline conditions that no room is left for improvement by the additional administration of WB platforms. Future studies may be performed with less rapidly fermentable carbohydrates, such as arabinoxylanoligosaccharides (AXOS). These oligosaccharides consist of a xylose backbone that is substituted with arabinose residues. By varying the degree of polymerization (DP) and the degree of substitution (DS), the rate of fermentation can be modulated. Unfortunately, ^13^C-labeled AXOS derivatives are not commercially available. Second, the appearance of ^13^CO_2_ in the breath and of ^13^C-SCFA in the plasma, consistently occurred before arrival of the test meal in the colon, indicated by the appearance of ^1^^4^CO_2_ in the breath, suggesting that the fermentation of inulin had already started in the terminal ileum, where the bacterial density rapidly increases [30]. We used native inulin, which is a mixture of oligo- and polysaccharides, with a DP varying from 3 to 70 and an average DP of 25. The presence of short fructo-oligosaccharides most likely explains the early fermentation, as fermentation of short oligosaccharides proceeds more easily than that of longer chains [31]. Indeed, studies that use the increase in breath hydrogen excretion after administration of inulin as a marker of OCTT [26,32,33], generally use Raftiline HP^®^ (Beneo, Mannheim, Germany) as the substrate, which only contains the long chains of inulin. This, at least in part, physical disconnection between the site of fermentation (terminal ileum) and main location of bacteria (colon), might hamper stimulation of the carbohydrate fermentation by bacteria adhered to the WB fractions.

Finally, repeated administration, rather than a single dose of WB, might have been more efficient for inducing a more efficient bacterial ecosystem. Nevertheless, in vitro incubation of human fecal samples with insoluble WB, showed that within 24 h (no earlier time point was tested) WB was already colonized by subsets of bacteria, suggesting a rapid colonization of insoluble particles [19]. Few studies have evaluated the impact of insoluble particles on SCFA production in vivo. Administration of resistant starch (RS) to pigs for two weeks resulted in increased cecal concentrations of butyrate, whereas combined administration of RS and WB resulted in increased butyrate concentrations in the more distal parts of the colon and in feces, suggesting that the addition of WB distally shifted fermentation [34]. These results could not be explained by partial fermentation of WB, as WB alone did not significantly increase butyrate concentrations. Also in humans, a diet supplemented with WB and RS for three weeks resulted in higher fecal proportions of butyrate and lower propionate proportions than WB alone, whereas fecal SCFA concentrations after the WB diet were not different from the values recorded during the control diet [35]. These observations support our hypothesis of improved carbohydrate fermentation due to the colonization of WB particles. However, alternative mechanisms also need to be considered. Increased fecal SCFA may also be due to the fact that WB accelerates whole gut transit time [36]. Indeed, both in vitro fermentation studies and human studies, indicate that SCFA production is increased with short transit time, and that longer transit times are associated with a shift from carbohydrate to protein fermentation [37,38,39]. It is important to note that fecal SCFA concentrations are the net result of production and absorption, and are difficult to relate to plasma SCFA.

Finally, we observed large inter- and intra-individual variations in plasma SCFA concentrations, which may have hampered detection of subtle changes in plasma SCFA due to WB intervention. Such large variations have also been reported in previous studies measuring SCFA [40,41,42] and may be due to variability in intestinal microbiota composition, colonic SCFA absorption and metabolism, and SCFA metabolism in the liver.

## 5. Conclusions

In this study, we showed that fermentation of a readily fermentable substrate results in increased plasma SCFA for about 8 h, suggesting that a sustained increase in plasma SCFA concentrations can be achieved when a moderate dose of fermentable carbohydrate is administered three times per day. Nevertheless, the addition of a single dose of different WB fractions did not further increase either the fermentation of the readily fermentable inulin, or cross-feeding between gut bacteria.
